# Body Iron Stores and Metabolic Syndrome in Adults Free of Cardiometabolic Disease From Southwestern Colombia

**DOI:** 10.1007/s12011-026-05056-y

**Published:** 2026-03-19

**Authors:** Milton Fabian Suárez-Ortegón, José Guillermo Ortega-Ávila, Diana María Caicedo-Borrero, Mildrey Mosquera

**Affiliations:** 1 Department of Food and Nutrition, Faculty of Health Sciences, Pontificia Universidad Javeriana Seccional Cali, Cali, Colombia; 2 Department of Basic Sciences, Faculty of Health Sciences, Pontificia Universidad Javeriana Seccional Cali, Cali, Colombia; 3 Department of Public Health and Epidemiology, Faculty of Health Sciences, Pontificia Universidad Javeriana Seccional Cali, Cali, Colombia; 4 Nutrition Group, Universidad del Valle, Cali, Colombia; 5 Department of Physiological Sciences, Universidad del Valle, Cali, Colombia; 6 Pontificia Universidad Javeriana Seccional Cali, Calle 18 No. 118-250, Cali, Colombia

**Keywords:** Metabolic Syndrome, Body Iron Stores, Ferritin, Cardiometabolic Risk, Cholesterol

## Abstract

Evidence linking serum ferritin (SF) with metabolic syndrome (MetS) remains inconsistent by sex. Data from Latin American populations are lacking, and adults free of cardiometabolic disease remain understudied. We examined whether SF was independently associated with MetS (harmonized definition) in adults from Cali, Colombia. In this cross-sectional study of 245 adults (123 women) without type 2 diabetes or cardiovascular disease, SF was the exposure, and outcomes included categorical MetS, a continuous MetS z-score, individual MetS components, and total and LDL cholesterol (LDL-C). Sex-stratified logistic and linear regressions were performed, unadjusted and progressively adjusted for age, lifestyle factors, C-reactive protein, and BMI. MetS prevalence was 24.6% in men and 12.2% in women; overweight/obesity prevalence was 64.1% (men 68.9%, women 59.3%). In men, each 1-SD increase in SF was associated with a higher MetS z-score across models (β [95% CI] 0.82 [0.39–1.26]; *p* < 0.001), whereas the association with categorical MetS was attenuated after adjustment for C-reactive protein and BMI. In women, SF was not independently associated with MetS. By components, higher SF related to higher fasting glucose and triglycerides in men, and to lower HDL-C in both sexes. SF was associated with LDL-C only in women. In men, SF increased with the number of MetS components, including one or two versus none (*p* = 0.043). In conclusion, in this Latin American sample free of cardiometabolic disease, SF was positively associated with MetS score in men, but not in women, among whom SF was consistently linked to LDL-C.

## Introduction

 Elevated body iron stores, measured by serum ferritin (SF) levels, are associated with an increased risk of type 2 diabetes (T2D) [1]. Ferritin is an intracellular protein essential for iron storage, with its synthesis regulated by intracellular iron levels. A portion of ferritin enters the bloodstream, serving as a biomarker for tissue iron storage [2]. Additionally, as an acute-phase reactant, ferritin production increases during infection and inflammation, likely as an adaptive mechanism to restrict iron availability to pathogens [3]. Although inflammation is a known risk factor for cardiometabolic diseases [4, 5], evidence suggests that SF’s association with T2D is independent of inflammatory processes, as meta-analyses indicate that adjustment for inflammatory markers, such as C-reactive protein (CRP), does not significantly alter this relationship [1, 6]. General adiposity (BMI) can also be considered a potential confounder because adipocytes store ferritin, excess adiposity promote subclinical inflammation, and also adiposity is a strong predictor of cardiometabolic disease [7, 8]. While systematic reviews and meta-analyses have shown that positive associations between SF and MetS generally remain significant after BMI adjustment, these associations may be strongly attenuated in populations with a high prevalence of obesity [9, 10]. Although the mechanisms by which iron excess contributes to T2D remain unclear, iron’s pro-oxidant properties may impair insulin signaling and/or disrupt cellular metabolism [9].

Metabolic syndrome (MetS), a well-recognized intermediate stage of risk for T2D, has also been positively associated with SF [10]. However, inconsistencies persist across subpopulations stratified by sex and menopausal status [11]. A notable limitation of the existing body of literature on iron stores and metabolic syndrome is the overrepresentation of Asian populations, since most studies have been conducted in that region [11]. Information on the ferritin-MetS association in Latin American individuals remains nonexistent to date. In Latin America, metabolic syndrome has emerged as a major public health issue. A recent systematic review and meta-analysis of studies in the region found that it is present in over 40% of adults, particularly in urban communities. In that analysis, the estimated prevalence of MetS in Colombia was 33.9%, while Mexico and Ecuador showed even higher prevalence, at 61% and 50%, respectively [12]. This cluster of metabolic and clinical alterations is not easily detected in clinical practice, as metabolic screening is often prioritized for individuals with obesity, while individuals who are not screened may experience worsening metabolic profiles and an increased risk of cardiometabolic diseases [13].

Additionally, studies on MetS and SF often do not evaluate additional cardiometabolic and atherogenic risk factors, such as cholesterol biomarkers, in relation to SF. Moreover, individuals classified as not having MetS may still have considerable cardiometabolic risk if they present one or two MetS components; such risk may also be similarly related to SF, and thus, alternative analytical approaches for the MetS outcome could be helpful to detect associations.

In the light of the above, we conducted a study to evaluate the association of SF with cardiometabolic risk, that included Mets, its individual criteria, and cholesterol biomarkers markers, and approaching MetS as both a categorical variable and as continuous score, in a group of adults from Southwest Colombia without T2D or cardiovascular disease.

## Methods

### Study Population

This study used data from a cross-sectional research project that investigated the association between iron status, insulin resistance, and diabetes. Participants were originally selected on a voluntary basis from three institutions and one supermarket chain in Cali, Colombia. The recruitment sites were: the Comfenalco Clinic (a private clinic in the city), Universidad del Valle (a public university), the Cali Health Department (the city’s local health authority), and La 14 Supermarket (a supermarket chain in southwestern Colombia).

### Sample Size and Recruitment

The original study aimed to compare levels of iron biomarkers between individuals with type 2 diabetes and apparently healthy individuals. The required sample size was estimated using ferritin concentration as the primary parameter, assuming that approximately 40% of individuals with type 2 diabetes would present elevated ferritin levels (clinical thresholds: >300 µg/L in men and > 150 µg/L in women) [14], with 90% reliability and statistical power. The target sample obtained consisted of 65 individuals with type 2 diabetes and 245 without diabetes. Recruitment was non-probabilistic and conducted from mid-2009 to the end of 2010 through advertisements describing the study objectives at the institutions mentioned above, which were selected for convenience. For the present analysis of serum ferritin (SF) and metabolic syndrome (MetS), we included only the group of individuals without diabetes (*n* = 245; 122 men and 123 women) aged 25–64 years. Participants with diabetes were not included in this analysis because MetS is highly prevalent in this population, which is inconsistent with the objective of examining the association between SF and MetS among individuals free of cardiometabolic disease.

### Exclusion Criteria

In the original project, to obtain an eligible apparently healthy sample of individuals without diabetes and to minimize confounding factors that could influence serum ferritin, lipid profile, and glucose levels, the following exclusion criteria were applied based on self-report by prospective participants: clinically significant hepatic, neurological, or endocrine disorders; cardiometabolic diseases (diabetes and cardiovascular disease, except type I obesity) or other major systemic conditions; smoking; regular use of multivitamin or vitamin supplements (≥ 2 days per week during the previous six months); ongoing pharmacological treatment for lipid or glycemic control; and evidence of acute or chronic inflammatory or infectious diseases. Recruitment continued until the required sample size was reached; therefore, there was no non-response rate to report.

### Procedures

Participants were scheduled to attend the Department of Physiological Sciences at Universidad del Valle in a fasting state and were received in a waiting area, where they were informed about the study objectives and their role as prospective participants. Individuals who agreed to participate provided written informed consent. In the same waiting area, trained research assistants conducted a brief screening interview based on the predefined exclusion criteria. Individuals who met any exclusion criterion were not enrolled in the study and were provided with breakfast before leaving the facility.

Eligible participants then entered a large assessment room organized as a five-station circuit, including: (1) anthropometric measurements, (2) venous blood sampling, (3) blood pressure measurement, (4) dietary intake assessment, and (5) interview on family history of cardiometabolic disease. Participants could start at any available station and proceeded sequentially to the next available station until completing the entire circuit. The only restriction in the order of assessments was that blood pressure measurement had to be completed before blood sampling, in order to avoid potential anxiety-related effects of venipuncture on subsequent blood pressure readings. After completing all stations, participants were provided with breakfast before leaving the facility.

### Clinical Assessments

Trained research staff conducted all anthropometric and blood pressure measurements following standardized international protocols. With participants wearing light clothing and no shoes, body weight and height were measured. Weight was measured using calibrated digital scales (Tanita BC-1000, Tanita Corporation, Tokyo, Japan) that were routinely checked and adjusted according to manufacturer specifications [15, 16]. Standing height was measured to the nearest 0.1 cm using a non-stretchable measuring tape (seca GmbH & Co. KG, Hamburg, Germany) mounted vertically on a flat wall. Participants stood barefoot, upright, and in the Frankfurt plane, with heels together and the back of the head, scapulae, buttocks, and heels in contact with the wall [17, 18]. A rigid right-angle headpiece was used to mark the highest point of the head before reading the measurement [18]. This wall-based method has demonstrated high agreement with stadiometer measurements and is considered an acceptable surrogate when standard stadiometer equipment is not feasible in field or resource-limited settings [17]. The vertical alignment of the measuring setup was verified prior to data collection using a level, and the tape scale was checked against a standard ruler. Body mass index (BMI) was derived as weight divided by height squared (kg/m²) [19]. Waist circumference was measured using a non-stretchable flexible measuring tape (seca GmbH & Co. KG, Hamburg, Germany), positioned horizontally at the midpoint between the lowest rib and the iliac crest, with participants standing upright and at the end of a normal expiration, following standardized anthropometric guidelines. Measurements were taken with the tape in direct contact with the skin, without compressing soft tissue [15, 20]. For blood pressure measurement, two readings were taken via a digital sphygmomanometer (OMRON^®^). The first reading was taken after 15 min repose, and the second 10 min after the first reading. The second reading was used for the study analyses [21].

### Biochemical Analyses

Venous blood samples were collected by trained phlebotomists using a closed Vacutainer® system following standardized clinical procedures after an overnight fast. Samples were drawn into serum separator tubes and allowed to clot at room temperature before centrifugation. Blood specimens were centrifuged at 3,500 rpm for 15 min to separate serum, which was then aliquoted into cryovials to avoid repeated freeze–thaw cycles. Serum samples were stored at − 20 °C, and biochemical analyses were performed within one week of blood collection. All procedures followed routine clinical laboratory quality assurance protocols to ensure sample integrity and reliability of analyte measurements. The following biochemical markers were assessed: fasting glucose using colorimetric, enzymatic method with glucose oxidase (cat No. 11504, Biosystems®ฏ, Spain) triglycerides using colorimetric, enzymatic method with glycerophosphate oxidase (cat No. 11828, Biosystems®ฏ, Spain), total cholesterol using colorimetric, enzymatic method with cholesterol esterase and cholesterol oxidase (cat No. 11506, Biosystems®ฏ, Spain), and high-density lipoprotein cholesterol (HDL-C) using a two steps reaction, an enzymatic elimination of other lipoproteins in the sample follow by colorimetric, enzymatic method with cholesterol oxidase and cholesterol oxidase (cat No. 11557, Biosystems®ฏ, Spain). Insulin levels were determined through chemiluminescence assays (IMMULITE 1000, San José, CA), while high sensitivity C reactive protein (hs-CRP) and serum ferritin (cat no.31927 and 13934 respectively, Biosystems®ฏ, Spain) were quantified via latex-enhanced immunoturbidimetry. Briefly, during the test, the molecule of interest in the sample binds with the specific antibody to cause agglutination. The turbidity caused by agglutination is detected optically by chemistry analyzer. The change in absorbance is proportional to the level of the molecule of interest in the sample. The actual concentration was obtained by comparing with a calibration curve with known concentration. An automated analyzer (A-15, Biosystems®ฏ, Spain) was used for these assessments. Commercial quality control (QC) samples were included in all the runs (cat no. 18042 and No. 18043, for colorimetric assays and cat no. 31211 and no.31212, for turbidimetric assays, Biosystems ®, Spain). The QC samples were analyzed following the procedures described above. Then, several QC runs were performed to determine the reproducibility of the sample preparation and the stability of the analytical platform used until the analytical system was equilibrated. After that, the QC samples were analyzed for every 30 randomized samples. Runs were accepted when the variation coefficients (intra-assay and inter-assay) were under 2% for colorimetric assays and 5% for turbidimetric assays.​

Insulin resistance was calculated using the Homeostatic Model Assessment for Insulin Resistance (HOMA-IR) index, employing the formula: Fasting glucose (mmol/L)x Fasting insulin (mU/mL)/22.5 [22].

### Metabolic Syndrome

MetS was operationalized in two ways: as a traditional binary outcome based on the harmonized definition from the 2009 joint interim statement [23], and also as a continuous score [24]. MetS harmonized definition comprises the following components: Elevated blood glucose, defined as fasting glucose levels ≥ 100 mg/dL; Triglyceride levels exceeding 150 mg/dL; reduced high-density lipoprotein cholesterol (HDL-C) (< 40 mg/dL in men and < 50 mg/dL in women); systolic blood pressure (SBP) ≥ 130 mmHg and/or diastolic blood pressure (DBP) ≥ 85 mmHg: and increased waist circumference (WC, ≥ 90 cm in men and ≥ 80 cm in women). Because none of the participant received any pharmacological treatment for increased triglycerides, low HDL-C or elevated blood pressure, these were not complementary part of the criteria as originally the MetS definition states. The presence of three or more components defined MetS [23].

A standardized continuous variable for metabolic syndrome (MetS) was also generated by averaging the Z scores of its key components [24]: blood pressure, glucose, triglycerides, HDL cholesterol, and waist circumference. A higher value of MetS Z score indicates a worse cardiometabolic profile. Specific statistical details on the MetS Z calculation are provided in the statistical analysis section.

### Dietary Intake and Family History

Dietary intake was assessed using a 24-hour dietary recall method, utilizing the food composition table developed by Quintero and Escobar [25]. All available nutrients listed in the table were evaluated and total caloric intake was estimated. Additionally, a questionnaire (authoring tool) was administered by trained interviewers to collect personal data and family history of cardiometabolic diseases in first- and second-degree relatives (parents, siblings, aunts/uncles, and grandparents). Physical inactivity or sedentarism was defined as engaging in no physical activity (30 min of vigorous moderate intensity) per week. Postmenopausal status was self-reported in terms of permanent absence of menstruation.

### Statistical Analysis

Descriptive analyses were performed for the entire study population and stratified by sex. All continuous variables were described as median and interquartile range, regardless of distribution, and were compared between sex groups using the Mann–Whitney U test. The rationale for this harmonized description is that the median is appropriate for non-normal variables and is similar to the mean under normality. Categorical variables were described as frequency (percentage), and the χ² test was used to compare these variables between sex groups. Prior to the MetS Z-score calculation, the distribution of these MetS components was graphically verified, and for the case of triglyceride levels, this variable was log-normalized because its distribution was left-skewed. To derive a single Z score for blood pressure, the Z scores of SBP and DBP were averaged. Additionally, the HDL-C Z score was multiplied by -1 to align all MetS components in the same direction, ensuring a consistent positive association with cardiometabolic risk [21].

Linear regression was used to assess the association of the exposure SF with three types of outcomes: (1) MetS z-score; (2) each MetS component, in its continuous z-score (as used to calculate the MetS z-score); and (3) serum total cholesterol and LDL-C. Logistic regression was used to evaluate the association between SF and the categorical MetS (presence of ≥ 3 components). To improve statistical power, SF was log-transformed due to skewness and then standardized as an SF z-score; therefore, the regression estimates and odds ratios reflect a 1-SD increase in log-transformed ferritin. The regressions were conducted with progressive adjustments to account for the effect of particular covariates. Covariates were selected based on potential influence on the exposure and/or outcomes, including age (years), lifestyle variables [physical inactivity (no/yes), total caloric intake (kcal/day), family history of cardiometabolic disease (no/yes)], hs-CRP levels as inflammation marker, and BMI. β-coefficients and 95% confidence intervals were estimated for: unadjusted models; age- and lifestyle-adjusted models (Model 1); additionally, adjusted for hs-CRP (Model 2); and additionally, adjusted for BMI (Model 3). For the relationships between SF and specific MetS components, estimates are presented for unadjusted and fully adjusted models.

All regression analyses were stratified by sex given meaningful differences in body iron stores. For women, Model 1 additionally included postmenopausal status because, in the absence of menstruation, iron storage capacity increases. Analyses stratified by menopausal status were not feasible due to limited sample size.

Additionally, we conducted ANOVA (unadjusted) and ANCOVA (adjusted for the covariates listed above) with SF as the outcome and the number of MetS components as the exposure, to test whether SF differed across categories, including individuals with one or two MetS components. This variation was also evaluated graphically by depicting confidence intervals for SF means within each category of the number of MetS components.

All statistical analyses were conducted using Stata version 14.2.

## Results

### Characteristics of the Study Population

Table 1 presents the baseline characteristics of the study population. Men had substantially higher SF levels than women and showed a less favorable cardiometabolic profile overall. Compared to women, men exhibited greater central adiposity, higher blood pressure, triglycerides, fasting glucose, and insulin resistance, while women had higher HDL-C and hs-CRP concentrations. Lifestyle factors also differed by sex. Men reported higher energy intake, whereas alcohol consumption was more common among women. No significant differences were observed for reported physical activity or family history of cardiometabolic disease. The prevalence of metabolic syndrome was nearly twice as high in men compared to women, largely driven by higher frequencies of hypertriglyceridemia and elevated fasting glucose. No sex-specific differences were observed for increased waist circumference, low HDL-C, or elevated blood pressure. Prevalence of overweight including obesity prevalence was 64.1% (men 68.9%, women 59.3%).

**Table 1:** Description of study population

	Men	Women	*P value*
*n*	122	123	
Ferritin (µg/L)	181(128–269)	70 (25–125)	**< 0.001**
Age (years)	45 (39–50)	46 (41–52)	0.204
Postmenopausal status *n* (%)		51 (41.5)	
BMI (kg/mts^2^)	26 (24–28)	26 (23–28)	0.542
WC (cm)	85 (78.5–91)	75 (70–80)	< 0.001
Systolic blood pressure(mmHg)	119.5 (112–127)	109.5(101–119)	**< 0.001**
Diastolic blood pressure(mmHg)	74 (69-80.2)	71 (63–79)	**0.018**
Triglycerides(mg/dL)	163 (117.7–228)	106 (79–149)	**< 0.001**
Glucose(mg/dL)	90 (85–96)	86 (82–90)	**< 0.001**
HDL-C(mg/dL)	43.1 (38.1–49.7)	52 (44.5–61.9)	**< 0.001**
LDL-C(mg/dL)	112 (96–136)	119 (95.5-133.7)	0.393
Colesterol (mg/dL)	196 (175.8-216.8)	197 (170–219)	**0.994**
hs-CRP (mg/L)	1.4(1.1–1.9)	1.7 (1.2–3.1)	**0.002**
Insulin (mU/mL)	9.4 (5.9–13.9)	7.4(5.6–12.4)	**0.062**
HOMA-IR	2.02 (1.26–2.99)	1.47 (1.05–2.55)	**0.015**
Kilocalories/day	1987.4 (1699.5-2350.7)	1759.6 (1508.6-1940.8)	**< 0.001**
Exercise practice *n* (%)			
30 min ≥ 3 times/week	44 (36.1)	38 (30.9)	**0.120**
30 min < 3 times/week	26 (21.3)	17 (13.8)	
Never	52 (42.6)	68 (55.3)	
Alcohol consumption *n* (%)	45 (36.9)	79 (64.2)	**< 0.001**
Family history of CMD	106 (86.9)	111 (90.2)	**0.429**
MetS Z score	0.18 (-1.77, 1.71)	-0.63 (-2.02, 1.87)	*
MetS *n* (%)	30 (24.6)	15 (12.2)	**0.013**
MetS components *n* (%)			
Increased fasting glucose	21 (17.2)	8 (6.5)	**0.010**
Increased triglyceride level	68 (55.7)	30 (24.4)	**< 0.001**
Increased blood pressure	34 (27.9)	25 (20.3)	**0.181**
Increased WC	36 (29.5)	36 (29.3)	**0.999**
Low HDL-C	40 (32.8)	50 (40.7)	**0.233**

Data are mean ± standard deviation or median (interquartile range). BMI, body mass index. WC, waist circumference. HDL-C, HDL cholesterol. LDL-C, LDL cholesterol. hs-CRP, high sensitivity C reactive protein. HOMA-IR, homeostatic model assessment insulin resistance. * Differences by group do not apply because Z-scores were calculated separately for each sex. CMD, cardiometabolic disease

### Serum Ferritin and MetS

In men, higher SF levels were strongly associated with greater MetS score (Table 2). Each 1-SD increase in SF Z score was linked to a higher MetS Z score in the unadjusted model (β = 1.25; 95% CI: 0.73, 1.77; p < 0.001), and this association remained significant after adjustment for lifestyle covariates (β = 1.11; 95% CI: 0.57, 1.65; p < 0.001) and hs-CRP (β = 1.05; 95% CI: 0.51, 1.59; p < 0.001). After further adjustment for BMI, the effect was attenuated but persisted (β = 0.82; 95% CI: 0.39, 1.26; p < 0.001). For categorical MetS, SF was associated with increased odds in the unadjusted model (OR = 1.74; 95% CI: 1.11, 2.73; p = 0.016) and after partial adjustments, but the association did not remain statistically significant when hs-CRP levels and BMI were included (OR = 1.64; 95% CI: 0.95, 2.83; p = 0.070).

**Table 2:** Relationships of ferritin Z score values with metabolic syndrome Z score (MetS Z score) and metabolic syndrome (MetS, categorical variable)

	Z score log-serum ferritin			
	MetS Z score		MetS	
	Beta (95% CI)	P value	OR (95%CI)	P value
*Men*				
Unadjusted	1.25 (0.73, 1.77)	< 0.001	1.74 (1.11, 2.73)	0.016
Adjusted for model 1	1.11 (0.57, 1.65)	< 0.001	1.64 (1.02, 2.63)	0.040
Adjusted for model 2	1.05 (0.51, 1.59)	< 0.001	1.58 (0.98, 2.55)	0.060
Adjusted for model 3	0.82 (0.39, 1.26)	< 0.001	1.64 (0.95, 2.83)	0.070
*Women*				
Unadjusted	1.13 (0.62, 1.65)	< 0.001	2.14 (0.99, 4.63)	0.051
Adjusted for model 1	0.90 (0.30, 1.49)	0.003	2.21 (0.90, 5.43)	0.083
Adjusted for model 2	0.49 (-0.11, 1.09)	0.111	1.63 (0.62, 4.23)	0.315
Adjusted for model 3	0.31 (-0.20, 0.83)	0.238	1.68 (0.60, 4.66)	0.318

Model 1: Adjusted for: age, postmenopausal status (for women), alcohol intake (no/yes), practice of exercise activity per week (≥ 3 times/ < 3 times/never), kilocalories/day, family history of cardiometabolic disease (no/yes). Model 2: Model 1 plus adjustment for hs-CRP level. Model 3: Model 2 plus adjustment for BMI.Beta, Beta coefficient. OR, Odds ratio. CI, confidence interval

Among women, SF was also positively associated with the MetS Z score in unadjusted analyses (β = 1.13; 95% CI: 0.62, 1.65; p < 0.001) and after lifestyle adjustment (β = 0.90; 95% CI: 0.30, 1.49; p = 0.003) (Table 2). However, the relationship was weakened and lost significance when hs-CRP (β = 0.49; 95% CI: − 0.11, 1.09; p = 0.111) and BMI (β = 0.31; 95% CI: − 0.20, 0.83; p = 0.238) were added to the models. In women, no significant associations between SF and categorical MetS were observed in unadjusted or adjusted models.

### Serum Ferritin and MetS Components

As shown in Table 3, higher SF levels were associated with several adverse cardiometabolic traits. In men, SF was independently related to higher fasting glucose (β = 0.19; 95% CI: 0.003, 0.38) and triglycerides (β = 0.19; 95% CI: 0.01, 0.38), and inversely associated with HDL-C (β = − 0.22; 95% CI: − 0.41, − 0.04). In women, the main associations were with lipid parameters: SF was inversely related to HDL-C (β = − 0.23; 95% CI: − 0.45, − 0.02) and positively associated with LDL-C (β = 0.37; 95% CI: 0.16, 0.57). For categorical MetS components´s outcomes, men with higher SF had increased odds of elevated fasting glucose (p = 0.014), while in women, SF was consistently associated with low HDL-C (OR = 1.15; p = 0.005), and additionally with increased WC (OR = 2.67, p = 0.034).

**Table 3:** Relationships between serum ferritin Z score values, MetS-related variables and cholesterol biomarkers

	log-ferritin Z score							
	Men				Women			
	Unadjusted		Adjusted*		Unadjusted		Adjusted*	
	Beta (95% CI)	P value	Beta (95% CI)	P value	Beta (95% CI)	P value	Beta (95% CI)	P value
*MetS components Z scores (continuous)*								
WC Z score	0.24 (0.08, 0.44)	0.004	0.05 (-0.02, 0.13)	0.173	0.34 (0.17, 0.50)	< 0.001	0.06 (-0.04, 0.17)	0.213
Glucose Z score	0.26 (0.09, 0.43)	0.003	0.19 (0.003, 0.38)	0.046	0.21 (0.04, 0.39)	0.016	-0.05 (-0.27, 0.15)	0.584
HDL-C Z score	-0.27 (-0.44, -0.10)	0.002	-0.22 (-0.41, -0.04)	0.013	-0.22 (-0.40, -0.04)	0.012	-0.23 (-0.45, -0.02)	0.027
Log-Triglycerides Z score	0.26 (0.09, 0.44)	0.003	0.19 (0.013–0.38)	0.035	0.29 (0.12, 0.46)	0.001	0.19 (-0.02, 0.41)	0.082
Blood pressure Z score	0.24 (0.08, 0.44)	0.004	0.15 (-0.02, 0.32)	0.083	0.05 (-0.11, 0.22)	0.499	-0.14 (-0.33, -0.05)	0.153
*Cholesterol biomarkers*								
Total cholesterol (mg/dL)	0.008 (-0.17, 0.18)	0.929	0.04 (-0.24, 0.15)	0.652	0.25 (0.08, 0.43)	0.004	0.19 (-0.02, 0.42)	0.078
LDL-C(mg/dL)	-0.05 (-0.24, 0.12)	0.539	-0.15 (-0.31, 0.08)	0.250	0.35 (0.18, 0.51)	< 0.001	0.37 (0.16, 0.57)	< 0.001
	OR (95%CI)	P value	OR (95%CI)	P value	OR (95%CI)	P value	OR (95%CI)	P value
*MetS components (categorical)*								
Increased WC	1.24 (0.83, 1.85)	0.289	0.87 (0.31, 2.41)	0.793	2.31 (1.34, 3.98)	0.003	2.67 (1.07, 6.63)	0.034
High fasting glucose	2.18 (1.29, 3.69)	0.004	2.12 (1.16, 3.86)	0.014	3.77 (1.04, 13.67)	0.043	0.99 (0.05, 18.67)	0.995
Low HDL-C	1.38 (0.93, 2.04)	0.105	1.29 (0.84, 1.97)	0.236	1.63 (1.08, 2.46)	0.019	2.15 (1.24, 3.73)	0.006
High Triglycerides	1.64 (1.10, 2.42)	0.013	1.46 (0.95, 2.24)	0.081	1.47 (0.92, 2.37)	0.106	1.35 (0.72, 2.50)	0.340
Increased blood pressure	1.57 (1.03, 2.39)	0.034	1.47 (0.92, 2.35)	0.103	1.07 (0.68, 1.68)	0.753	0.88 (0.47, 1.67)	0.712

* Adjusted for: age, postmenopausal status (for women), alcohol intake (no/yes), practice of exercise activity per week (≥ 3 times/ < 3 times/never), kilocalories/day, family history of cardiometabolic disease (no/yes). CRP levels (log-transformed), and BMI.Beta, Beta coefficient. OR, Odds ratio. CI, confidence interval

**Fig. 1:**
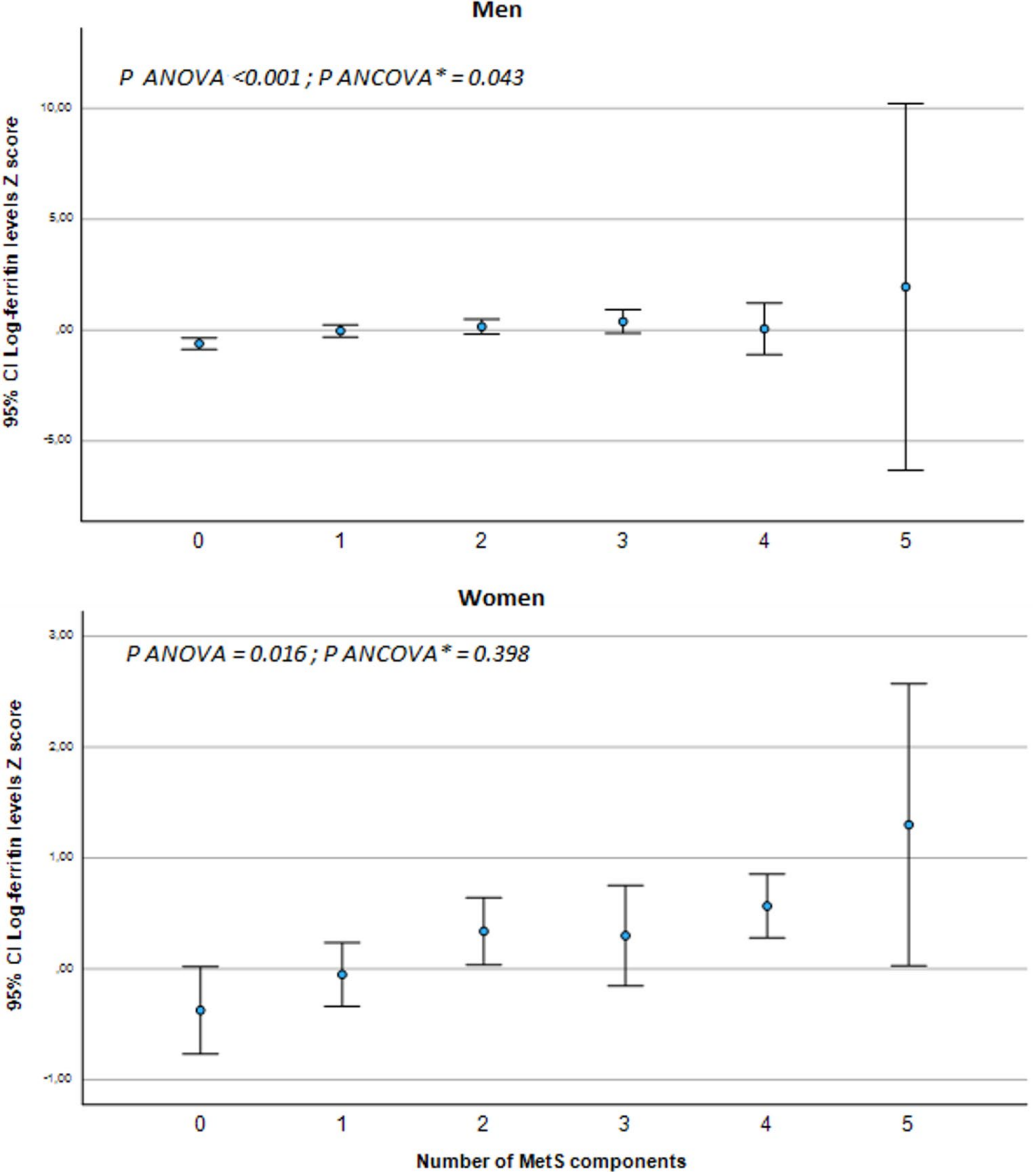
Serum ferritin levels (Z score of log-transformed values) by number of metabolic syndrome (MetS) components in men and women. Data are means and their standard error. Differences in ferritin levels estimated via ANOVA (unadjusted) and ANCOVA. *adjusted for age, postmenopausal status (for women), alcohol intake (no/yes), practice of exercise activity per week (≥3 times/ < 3 times/never), kilocalories/day, family history of cardiometabolic disease (no/yes), CRP levels (log-transformed), and BMI. P, p value

When the number of MetS components and SF were treated as the independent and dependent variables, respectively (Fig. 1), mean SF in men was significantly higher with increasing numbers of MetS components, including for one or two components versus none (Fig. 1A). In women, the same trend was observed, but statistical significance was lost after adjustment for covariates (Fig. 1B).

## Discussion

In this cross-sectional study of the relationship between serum ferritin (SF) and metabolic syndrome (MetS) in individuals from a city in southwest Colombia, SF was significantly associated with MetS when MetS was approached as a continuous risk score, and only in men. When MetS was used as a categorical variable (≥ 3 MetS components), its association with SF was specifically attenuated after adjusting for BMI. In women, the SF–MetS z-score and SF–MetS associations lost statistical significance after basic adjustment for age and lifestyle covariates. However, only in women was an association observed between SF and LDL-C (continuous or categorical), a marker of atherosclerotic risk. Our findings are the first described for SF and MetS in a group of Latin American individuals and provide evidence that the pattern of association between SF and cardiometabolic risk varies by geographic region and by sex.

In the present study, we found in men that higher SF levels were associated with a higher MetS z-score, both in unadjusted analyses and after adjustment for age, lifestyle covariates, hs-CRP, and BMI. This sex-specific relationship might be explained by the fact that men have higher iron stores than women, and higher SF concentrations may lead to stronger associations with cardiometabolic risk. No previous adult studies on SF and cardiometabolic risk have approached MetS as a continuous risk score, and the replicability of this finding warrants confirmation in future work. Although SF was also significantly associated with categorical MetS across different adjustment models, it did not remain statistically significant when BMI was included as a covariate. This discrepancy, depending on whether MetS is modeled as a continuous vs. categorical outcome, has several possible explanations. Analytically, compared with a categorical outcome, a continuous outcome increases statistical power to detect associations; consistently, a larger sample of men might have allowed identification of an independent relationship between SF and categorical MetS. However, although a meta-analysis on SF and MetS reported an overall significant association, it also highlighted that some larger studies reported non-significant independent associations [26, 27] in both sexes, or in men [28–31]. Moreover, the same meta-analysis, via meta-regression, found that BMI and hepatic-injury markers produced the greatest attenuation of the SF–MetS association. Excess adiposity can confound the SF–MetS link because adipocytes are a source of ferritin and because fat-driven inflammation increases cardiometabolic risk [7, 32]. This would affect associations in populations in which overweight/obesity is highly prevalent. In agreement with this, a recent population-based study from the Shetland Islands (UK), in which overweight including obesity prevalence was 70% reported a positive association, by comparing extreme SF quartiles, between higher SF and a higher likelihood of MetS in men and postmenopausal women that became non-significant after adjustment for BMI [33]. In our study population, overweight including obesity was present in 64.1% of individuals; according to the 2015 Colombian National Nutritional Survey, 56.2% of Colombian adults have overweight or obesity [34].

A potential factor affecting comparability across studies may be the laboratory method used for ferritin determination, although the magnitude of this influence should be interpreted with caution. Two systematic reviews and meta-analyses reported trends toward heterogeneity in the SF–MetS association, with stronger associations observed in studies employing immunoradiometric assays compared with other analytical methods [10, 11]. However, these meta-analytic comparisons were not stratified by sex or menopausal status because statistical power would be substantially reduced, Additionally, immunoradiometric, ELISA-based, chemiluminescence-based, and immunoturbidimetric (agglutination) assays have been shown to be comparable [35, 36], and all are recommended by the World Health Organization for serum ferritin measurement [37].

We also found that, in men, the greater the number of MetS components, the higher the SF. This association was statistically significant even after adjustment for covariates, including BMI. Again, this highlights that using a continuous outcome (in this case, SF) can facilitate detection of independent relationships. It may also imply that individuals with one or two MetS components provide additional evidence for a significant relationship between SF and cardiometabolic risk, which could be overlooked when analyses rely on the more stringent binary outcome of ≥ 3 components to define MetS.

Taken together, the findings from all analytical approaches in our study indicate that a link between MetS and SF was evident in men but not in women. This may be related to the higher iron levels typically observed in men compared with women. Nevertheless, a meta-regression of studies on SF and MetS did not find a significant difference in the association across sex or menopausal-status subgroups [11]. In men, at the level of specific MetS components, our results are consistent with meta-analyzed findings showing that higher triglycerides and higher glucose (components commonly related to SF) support, to a large extent, the SF–MetS link [11]. Low HDL-C was also associated with SF in both men and women in our study.

Although a SF-MetS link was not found in women, there was clear pattern of specific independent association between increased total cholesterol and LDL-C only in women. This could imply that insulin resistance, a strong promoter behind MetS onset, would be more related to SF in men, and atherogenic factors would be more related to SF in women. Evidence on a relationship between SF, as a proxy for body iron stores, and the lipid profile suggests an association with atherogenic dyslipidemias, characterized by elevated LDL-C and triglycerides and reduced HDL-C levels. Several studies have reported positive correlations between ferritin and total cholesterol or LDL-C concentrations [38–42] and triglycerides [40–42], as well as negative correlations with HDL-C [38, 40, 41]. However, few studies have conducted robust adjustments to describe the associations between SF and lipid profile variables [38, 40, 41, 43] and reported sex-stratified relationships [40, 41]. Ramakrishnan et al. exclusively evaluated women from NHANES and found a significant positive relationship between serum ferritin (SF) and a composite variable integrating all lipid profile biomarkers together with glucose, BMI, and blood pressure; this association was independent of age, prevalent infection, recent blood donation, and recent treatment for anemia [43]. However, the integrated outcome did not allow conclusions about biomarker-specific relationships. In addition, Alissa et al. specifically studied a male population from Saudi Arabia comprising individuals with and without coronary disease; in multivariable models, serum total cholesterol and dietary cholesterol intake were the only significant predictors of SF after testing several dietary and lifestyle covariates [38]. Meanwhile, Kim et al. and Jin et al. reported discrepant sex-specific findings in Korean adolescents (10–19 years) and Tajik adults in China, respectively. Whereas Kim et al. found a significant independent association between SF and total cholesterol and LDL cholesterol in males but not females, Jin et al. reported trends for the same associations only in women [40, 41]. This discrepancy might relate to the markedly different age ranges between the studies; growth and development during adolescence may influence or confound a transitional link between SF and LDL cholesterol. Our findings of a specific relationship between SF, total cholesterol, and LDL cholesterol in adult women agree with the study by Jin et al. Although both Jin et al.’s study and ours were conducted in adults, Jin et al. did not report inclusion and exclusion criteria, which limits the assessment of sample comparability to support the similar associations observed [40].

There are potential mechanisms that may explain either independent or non-independent positive associations between SF and serum total cholesterol or LDL-C. Iron excess in cells may affect cholesterol synthesis and/or handling via: (1) activation of the SREBP-2 transcription factor, which upregulates cholesterol-biosynthetic genes (e.g., HMGCR) [44, 45]; (2) oxidative stress–mediated upregulation of microsomal triglyceride transfer protein, promoting higher VLDL secretion and subsequent LDL-C circulation [46, 47]; (3) iron overload-promoted disruption of bile acid synthesis/efflux with increase hepatic cholesterol [48, 49]; and (4) inflammation, which increases ferritin levels alongside small, dense LDL and raises PCSK9 (via SREBP-2/HNF1α pathways), thereby degrading LDL receptors, reducing LDL clearance, and raising LDL-C [50]. This latter mechanism is unlikely to explain our findings, since the SF–LDL-C relationship in women remained significant after adjustment including CRP, and in men the unadjusted associations between SF and cholesterol markers, before the adjustment for CRP levels, were not statistically significant. However, larger epidemiological observational studies are needed to confirm potential sex differences in the link between iron and cholesterol metabolism.

### Limitations and Strengths

Several limitations have to be acknowledged. First, the cross-sectional nature of our study does not enable to infer causal relationships and may also imply potential reverse causality, since metabolic alterations could influence SF. Second, the 24-hour dietary recall was conducted for a single day, which may limit the accuracy of the estimates of the covariate of individual intake. Third, because participants were recruited using non-probabilistic sampling, the results may not be representative of the general adult population in Colombia and should be interpreted with caution and contrasted with future population-based studies involving individuals without chronic non-communicable diseases. Fourth, questionnaires used for information on physical activity and familial history cardiometabolic disease were authoring tools and not validated international questionnaires. Similarly, the information on the above variables was based on self-report, which may imply recall bias. However, in previous studies, these kinds of covariates have not shown to influence the SF-cardiometabolic risk relationship. Additionally, the attenuation of the ferritin–MetS (categorical) association after adjustment for BMI may have a partial technical explanation, since the sample size was modest and MetS is an outcome that may include, within its cluster of criteria, increased waist circumference, which is more closely related to the BMI covariate. However, as previously mentioned, even in studies with much larger sample sizes [33], BMI adjustment has reduced the strength and statistical significance of the SF–MetS association. Finally, residual confounding cannot be ruled out, as both ferritin and cardiometabolic markers may be influenced by multiple unknown pleiotropic metabolic effects, and also factors such as detailed menstrual blood loss patterns, liver function, genetic iron disorders, or specific dietary patterns were not measured in this study.

Conversely, a strength of our study lies in the recruitment of participants who were free of cardiometabolic disease and potential confounders, such as tobacco use, multivitamin supplementation, or regular medication intake. However, the exclusion for diseases was based on self-report, and thus, it is possible that some individuals included might have had cardiometabolic or systemic diseases without knowing it. The alternative use of MetS as a continuous variable represents another strength, since it allowed to contrast findings from MetS as categorical variable. Additionally, in our analysis, the concomitant evaluation of cholesterol-related cardiometabolic risk markers expanded and improved the characterization of the SF-cardiometabolic risk relationship.

### Conclusion

SF was cross-sectionally associated with MetS when assessed as a continuous score, but only in men; when MetS was modeled categorically, the association was attenuated after adjustment for BMI. In women, although associations with MetS were not significant, SF was consistently related to LDL-C, a marker of atherosclerotic risk. Particularly, longitudinal studies are needed to confirm a prospective link between elevated SF and incident high LDL-C, and to determine whether this relationship is sex-specific. These findings are the first reported in a Latin American population and complement systematically reviewed and meta-analyzed evidence from studies conducted predominantly in Asian cohorts.

## Data Availability

Data available on request from the authors.
